# The evolving potential for dementia prevention in Switzerland: population attributable fractions of risk factors over time

**DOI:** 10.1002/alz70860_100771

**Published:** 2025-12-23

**Authors:** Ozge Sayin, Augusto J. Mendes, Federica Ribaldi, Giovanni B. Frisoni

**Affiliations:** ^1^ Geneva Memory Center, Department of Rehabilitation and Geriatrics, Geneva University Hospitals, Geneva, Geneva, Switzerland; ^2^ Laboratory of Neuroimaging of Aging (LANVIE), University of Geneva, Geneva, Switzerland; ^3^ Laboratory of Neuroimaging of Aging, University of Geneva, Geneva, Switzerland

## Abstract

**Background:**

It is estimated that modifiable risk factors (RFs) account for approximately 45% of dementia cases worldwide. While several studies have examined the extent to which these RFs contribute to dementia incidence in other regions, such as Brazil, Denmark, China, USA, Switzerland's specific context has not been investigated. This is of particular interest because recently it has been shown that dementia incidence is declining in many high‐income countries. Therefore, we aimed to calculate the population attributable fraction (PAF) in the Swiss population who have access to high‐quality health care system to inform effective dementia prevention strategies.

**Method:**

We will use the Swiss National Health Survey, a nationally representative dataset, in which potentially modifiable dementia RFs were collected every five years from 1992 to 2022. The PAF for each risk factor will be estimated based on its prevalence and the associated relative risks from prior meta‐analyses. We will adjust for the communality between RFs and calculate overall weighted PAF, focusing on variations across cantons and over time.

**Result:**

We expect that the PAF for dementia in Switzerland will show how the potentially modifiable RFs have contributed across the years to dementia incidence. We anticipate that the overall PAF might be lower than in low‐income countries, reflecting Switzerland's high quality of health care system. In addition, due to the lifestyle changes in Switzerland, we expect to see a decrease in weighted PAF between 1992 and 2022. The harmonization of the data across years is ongoing and the results will be presented at AAIC in July 2025.

**Conclusion:**

This study is essential for understanding how potentially modifiable factors contribute to dementia risk in Switzerland. By identifying the contribution of the RFs, we will reveal the potential for dementia prevention in the Swiss context. These findings will offer crucial evidence to guide public health policies and targeted interventions, helping to reduce the burden of dementia in Switzerland.

